# Associations between Motor Competence, Physical Self-Perception and Autonomous Motivation for Physical Activity in Children

**DOI:** 10.3390/sports8090120

**Published:** 2020-09-01

**Authors:** Ole Kristian Ensrud-Skraastad, Monika Haga

**Affiliations:** 1 Department of Neuromedicine and Movement Science, NTNU Norwegian University of Science and Technology, 7491 Trondheim, Norway; okskraastad@hotmail.com; 2 Department of Teacher Education, NTNU Norwegian University of Science and Technology, 7491 Trondheim, Norway

**Keywords:** motor skills, athletic competence, self-concept, intrinsic motivation, self-determination theory

## Abstract

Research indicates that children and adolescents gradually participate less in physical activity with age. Several factors are associated with children’s physical activity levels, such as motor performance, self-perception of athletic competence and motivation to physical activity. To gain a better understanding of the factors of importance for behavior related to an active lifestyle, the purpose of this study was to investigate the association between motor competence, physical self-perception and autonomous motivation and to examine to what extent this association may vary by sex. The sample consisted of 101 children, whose average age was 11.7 years (SD = 0.57), 53 boys and 48 girls. All subjects were measured on motor competence, physical self-perception and autonomous motivation for physical activity. The results indicate a low positive relationship between motor competence and physical self-perception for the entire sample and among girls. There is also a significant correlation between autonomous motivation and physical self-perception. No significant correlations were found between autonomous motivation and motor competence. The association between physical self-perception and autonomous motivation suggests that psychological factors play an important role in children’s participation in physical activity.

## 1. Introduction

Despite increased knowledge about the positive health effects of physical activity, sedentary activities and low physical activity levels are an increasing challenge throughout the life span [1]. Most evidence suggests that there is a clear decline in activity levels with age throughout adolescence [2,3]. An important focus of research on health and quality of life among children and adolescents has been to identify the physical, psychological and social factors related to participation in physical activity in this age group.

There is evidence for a reciprocal association between motor competence and physical activity [4,5,6,7]. Motor competence can be understood as a person’s ability to perform various motor actions, including the coordination of fine and gross motor skills, and these are necessary to master activities in everyday life, including play and physical activity [8,9]. Possessing certain motor skills allows the child to participate in varied physical activities, which in turn facilitates versatile movement experiences [10]. At the same time, stimuli and experience through participation in physical activity give rise to both quantitative and qualitative changes in motor development, that is, learning new motor skills and increasing the quality of movement in skills already learned [11,12]. In recent years, a decline in motor skills in children and adolescents has been reported [13,14,15]. A strong correlation has also been found between motor competence and components of physical fitness, including body weight status, endurance and muscle strength [13,16,17].

Girls often perform better than boys on manual/fine motor skills [18,19,20,21,22], while boys perform better in ball skills [19,20,23,24]. In terms of balance, less differences are found between sex, although there is a tendency that girls outperform boys. For children aged four years, total scores on motor competence were higher in girls compared to boys [22]. The girls’ lead over boys in fine motor manual skills was no longer found after puberty [18].

In their review article, Babic et al. [25] report a significant relationship between participation in physical activity and physical self-concept and its various subdomains in children and adolescents. Self-perception describes a person’s perceptions, judgments and expectations of themselves in different situations in domains such as physical appearance, social acceptance, physical- and academic competence [26]. Self-perception provides the basis for a person’s feelings, motives and behavior. Harter’s theory of self-concept [26,27,28,29] is based on the effectance motivation theory by White [30]. White’s theory emphasized that motivation to gain competence not only came from within or was innate, but also evolved in interaction with the environment [30]. Harter [27,28] found that children as young as four years of age showed different motivation depending on their interests. Harter [31] developed a multidimensional approach to competence motivation with six different domains of competence: academic competence, social acceptance, physical competence, physical appearance, behavioral conduct and global self-worth. Physical self-perception concerns judgement of both competence in subdomains such as physical activity, sports, active play and motor skills. According to Estevan and Barnett [32] physical self-perception also contains the subdomain perceived motor competence, which is related to perceived competence in fundamental movement skills such as stability, object control and locomotion. Experiences and whether one succeeds or fails with one’s coping attempts decide whether the individual receives positive or negative mastery [27]. The theory of competence motivation shows that children are attracted to situations where they feel they are the masters and avoid situations where they feel that they do not have competence [33].

Boys generally have higher physical self-perception and perception of physical appearance than girls [34,35,36,37,38,39,40]. A higher physical self-perception may be a consequence of higher actual competence in the physical domain found among boys compared to girls [41], however, it is also been pointed out that higher self-perception in boys may be because they tend to overestimate their own competence in the physical domain [4].

Self-determination theory (SDT) has been widely used to understand the motivation processes associated with health behavior and especially motivation for physical activity [42,43]. In SDT, motivation is described as the very starting point for what activates people, and there are basic biologic, psychological and social factors that activate, regulate and maintain behavior [44]. SDT is based on three fundamental psychological needs: autonomy/self-determination, competence and relatedness, which act as nutrients for motivation [45,46].

SDT has a qualitative approach to motivation, where different types of motivation are described from a qualitative scale. The types of motivation are based on various causes or goal attainments that form the basis of the activity. The catalyst in increasing the quality of motivation in the SDT theory is self-determination, also called autonomy [47]. SDT describes three motivational forms driven by self-determination: internal motivation, which is self-regulated; external motivation, which is regulated by external factors and amotivation; which is unregulated [48]. Internal motivation is regarded as the motivation of the highest quality because, in addition to giving a sense of voluntariness, it also gives emotions such as joy, satisfaction and exhilaration. External motivation, also referred to as controlled motivation, has lower quality because it uses external forms of pressure [47]. This pressure can be either punishment or reward, but anyway it is an external motivation. The individual’s autonomy is a deciding factor which influences whether one acts based on intrinsic values, drives and needs, rather than external factors such as social expectations or rewards. According to SDT, motivation for physical activity can be regulated along a continuum based on the degree to which the behavior is autonomous. Six types of behavioral regulations are proposed in SDT; intrinsic motivation, integrated regulation, identified regulation, introjected regulation, external regulation and amotivation. Integrated motivation is a complex type of motivation not usually found in children [46], while amotivation assesses the presence vs. absence of motivation rather than the level of self-determination. Intrinsic motivation is considered the most autonomous form of motivation while identified, introjected and external regulations are extrinsic forms of motivation. Intrinsic, identified, introjected and external motivational regulations have been adapted to be appropriate for the measurement of children’s motivation to physical activity [46].

Motivation is complex, and there are obvious gradations of external or internal motivation that fall between these extremes [49]. In terms of participation in physical activity, it was envisaged that external and internal motivation were too extreme on their own and that a continuum had to exist between them that provided a more accurate description of motivation [49,50]. Autonomy is often seen in the context of self-regulation. It is autonomy and self-regulation that determine a person’s initiation, coordination and behavior [51]. Autonomous motivation means that the person has a holistic idea of what the activity entails in terms of advantages and disadvantages, in several ways. This includes future plans and goals, which give participation in an activity at a given time a long-term significance. This is especially relevant in integrated regulation. It lies close to intrinsic motivation, but may, for example, underlie external factors that can be part of a long-term goal [45].

Autonomous motivation also has a close relationship to self-perception. Self-confidence occurs when participation in activities is perceived as self-determined and is integral to the true self, as opposed to aspects that are imposed or based on external stimuli [50]. Research shows that autonomous motivation in relation to physical activity has significant benefits. Students with intrinsic motivation in physical education had a more positive attitude toward physical activity in their leisure time [52,53], better concentration in physical education [53], higher effort in training contexts [52], higher interest in physical activities [54] and willingness to try challenging physical activities [53]. There is also a tendency for declining motivation at the various motivation levels with age [55,56,57,58,59]. Boys are found to have higher levels of motivation over time in childhood and adolescence than girls [55,58,59]. In the age range 10–11 years, boys evaluate that they have better skills than girls, while having a higher level of participation and pleasure in physical activity than girls [55]. In this context, De Mester et al. [60] argue that a personal-centered approach to identify different profiles of perceived competence and actual motor competence at the personal level could be important as different profiles seem to influence motivation to physical activity in different ways. A recent study analyzing the relationship among motivation, actual and perceived motor competence in early adolescents found curvilinear association between perceived competence and motivation [61]. This means the difference in motivation is larger between children with very low and low self-perception compared to children with high or very high self-perception. Actually, different profiles are found among adolescents, and a high perceived competence seems to play a key role in motivation and engagement in physical activity despite actual motor competence [60]. Moreover, Kalaja et al. [62] found that among students at the age of 13, students within the profiles “high motor skills/high autonomous motivation” were more physically active compared to students within the profiles “low motor skills/low autonomous motivation” and “high motor skills/low autonomous motivation”.

A potential strategy to ensure that children and young people maintain an active lifestyle and a lifelong joy in movement is to foster motivation for physical activity. Based on this, it becomes important to gain increased understanding about the associations between factors relevant to engagement in physical activity. In this context, motor competence has received less attention a possible underlying mechanism of behavior related to physical activity among children. In addition, a lack of motivational regulations validated questionnaires for children has led to less attention on children’s motivational regulation related to physical activity and sports. To gain more insight into the associations between judgement of competence in physical activity engagement, i.e., physical self-perception, actual motor competence and motivational regulations would further expand our understanding of relevance to physical activity behavior [61].

The purpose of this study was therefore to investigate the associations between motor competence, physical self-perception and motivational regulation in children aged 11 and 12 years and the extent to which this association varies with sex.

## 2. Methods

### 2.1. Participants

The sample consisted of 101 students recruited from schools from one county in Norway (11–12 years, M 11.7 years, SD 0.57 years). There were 53 boys and 48 girls. A total of 167 children were recruited to the study, giving a respondent rate of 60.5%. A random selection of schools was drawn from the nearest schools in the region. All the schools were recruited from public schools in the same county and in municipalities close to each other with similar demographics. The schools belong to downtown areas of smaller cities or towns. The four schools that first signalized they wanted to participate in the study became the study schools. The participating schools were considered to be “average” schools regarding sociocultural- and economic conditions. One can assume that there are no cultural differences between the different schools regarding the variables in focus. Both leisure-time activities, school curriculum, and sport participation for children and adolescents are similar in values and principles across these municipalities. In Norway, all schools adhere to the same curriculum in PE and similar hours is dedicated to PE across the country. Exclusion criteria at the individual level included: physical or cognitive conditions or injuries that could potentially interfere with the ability to carry out the testing.

### 2.2. Study Measures

#### 2.2.1. Autonomous Motivation for Physical Activity

Intrinsic, identified, introjected and external motivation related to physical activity in children was measured using an adapted scale based on Sebire et al., [46], which is developed from the behavioral regulation in exercise questionnaire (BREQ) [46,49]. BREQ has been adapted to be appropriate for the measurement of children’s motivation to physical activity [46]. The scale consisted of 12 items, 3 per motivation subscale. The adapted scale consisted of the stem “Boys and girls can be active by doing all sorts of things, for example walking, playing out or doing sports. The following pages have some reasons why you may be active. Please indicate how true each one is for you”. Items were preceded by “I am active because”. Three items each measured the four different types of motivational regulations; intrinsic (e.g., I like to be in physical activity), identified (e.g., It is important to me to do active things) introjected (e.g., When I’m not active I feel unhealthy) and external motivation (e.g., Other people push me to be active). Items were scored using a 5-point Likert scale (ranked from 1 = not true for me to 5 = very true for me) and an average score was summed for each of the four subcategories. Each subcategory gave a score between 3–15. The form was translated into Norwegian and a subject teacher in English contributed to the translation to ensure equivalence to the original form. Previous studies show satisfactory construct and concurrent validity of the adapted scale for motivation among schoolchildren with a Cronbach’s alpha coefficients for the motivation subscales of: intrinsic (α = 0.77), identified (α = 0.71), introjected (α = 0.59) and external (α = 0.71) [46].

#### 2.2.2. The Self-Perception Profile for Children (SPPC)

The self-perception profile for children (SPPC) [28,31] is a questionnaire designed to map children’s domain-specific judgement of their scholastic competence, social acceptance, athletic competence, physical appearance, behavioral conduct and global self-worth [31]. SPPC is considered one of the foremost instruments for clinical studies of children’s mental strengths [37] and has been found to be a valid and reliable way for children to assess self-perceptions [26,37]. Since this study wanted to look at physical self-perception, i.e., judgement of competence in physical activity, sports, active play and motor skills, it was important to use a measure that capture all these subdomains. As the aim of the study was to explore the association between athletic competence, actual motor competence and motivation for physical activity, a self-perception measure that contains judgments of a broad set of subdomains was chosen. Only the scale related to athletic competence from SPPC was used. In this study, the Norwegian translation was used [63]. The athletic competence subscale consists of six items, each of which consists of two opposite descriptions, e.g., “Some children prefer to look at other children play”, but “Other children prefer to join in the play rather that look at others”. First, the child must decide which statement best reflects them, and second, they decide if it is “really true” or “sort of true” for them. As each level has two grades, the scale gives a rating from 1–4, 4 indicates the perception of highest physical competence, while 1 indicates the lowest competence [26]. This format has demonstrated to be a good way to avoid receiving socially desired responses from the respondents [64]. The internal consistency of the SPPC scales are found to be satisfactory with Cronbach’s alphas between 0.73 and 0.81 [37].

#### 2.2.3. Test of Motor Competence (TMC)

Test of motor competence (TMC) [11] was used to measure motor competence. TMC consists of four tasks: two fine motor tasks, based on manual dexterity and eye–hand coordination and two gross motor tasks, based on dynamic balance. The performance measure was time to completion. Participants were given an exercise trial before the test trial itself began. TMC has an acceptable internal consistency of the tasks in the test battery, as all test tasks correlate positively with total scores (with correlations ranging from 0.48 to 0.64). The construct validity between the movement assessment battery for children (MABC) and TMC was 0.47 for ages 7–8 years and a test-retest reliability coefficient for placing bricks of 0.90, for building bricks of 0.75, for heel-to-toe walking of 0.94, for walking/running in slopes of 0.94 and total score of 0.87 [11].

Fine motor tasks: placing bricks (PB). Eighteen square-shaped Duplo™ bricks had to be placed on a Duplo™ board (3 × 6 bricks) as fast as possible. The child was seated at a table and given a practice run before the testing. The bricks were positioned in horizontal rows of three on the side of the active hand and the board was held firmly with the other hand. Both hands were tested.

Fine motor tasks: building bricks (BB). Twelve square-shaped Duplo™ bricks were used to build a “tower” as fast as possible. The child held one brick in one hand and one brick in the other. At a signal, the child assembled the bricks together, one after the other, until all 12 were put together to form a tower. Arms were not allowed to rest on table. The bricks were required to be held in the air at all times. The tasks were conducted with the child sitting at a table, and the time was stopped when the child released contact with the last brick. Two attempts were allowed, but only the best time was recorded.

Gross motor tasks: heel-to-toe walking (HTW). The child walked down a straight line (4.5-m long) as fast as possible, placing heel against toe in each step. Two attempts were allowed, but only the fastest time was recorded.

Gross motor tasks: walking/running in slopes (W/R). The child started at the starting point. At a signal, the participant walked/ran as fast as possible in a figure of eight around two marked lines (1-m wide). Line 1 was 1 m from the starting point and Line 2 was 5.5 m from the starting point. If the participant started to go on the right side of the Line 1, he/she would go to the left side of Line 2, turn around and go back on the right side of Line 2 and left side of Line 1 and over the starting point. The time was stopped when the child arrived back at the starting point. Participants freely chose in which direction they walked/ran. The children wore suitable shoes. Two trials were allowed, of which the fastest time was recorded.

### 2.3. Procedure

Information about the study and a written consent form were sent to parents. The study was approved by the Norwegian Center for Research Data (NSD), identification number 54,474. Before the start of testing and the questionnaires being published, common information was given to the participants about the project and which tests and questionnaires were to be used in an age-appropriate manner. The assessment of physical self-perception and motivation for physical activity took place in classrooms during school hours. To ensure that everyone understood each question, each section of the forms was read aloud to the entire group before proceeding to the next question.

The testing of motor skills was carried out in accordance with the manual of the test battery. The testing was done following surveys for the other variables. All the participants were tested one-by-one, with no spectators or other participants present. Oral encouragement and support were provided during the testing. Data were collected in the autumn 2017.

### 2.4. Data Reduction and Analysis

All statistics were carried out in IBM SPSS version 25 (IBM Corporation, New York, NY, USA). A total TMC score was calculated by transforming test item scores into standardized scores (z scores) using the overall sample mean. The total test score for each child was calculated as the average z-score on all test items successfully performed by the same participant. In addition to the z-score, descriptive results from the four motor tasks are presented. A Mann–Whitney *U*-test with Bonferroni’s correction was used to compare the differences between the sex in the different variables. The correlation analyses were performed with Spearman´s rho since the data were not normally distributed. Spearman’s rho was conducted in each sex separately. The following guidelines for effect strength were proposed: <0.2—may be considered trivial; 0.2–0.5—small; 0.5–0.7—moderate and >0.7—large [65]. Significance level was set at *p* ≤ 0.05.

## 3. Results

Means and standard deviations are shown in Table 1. The results show no differences between sex in motor competence, motivation and physical self-perception.

There was a low correlation between motor competence and physical self-perception for the entire sample (r = −0.242) (Table 2). The associations between motor skills and physical self-perception display sex differences. Table 3 shows that boys had a low correlation between physical self-perception and gross motor skills (r = −0.308), but no correlation with fine motor skills (r = 0.089) or total motor competence. Table 4 shows that girls had low correlation between total motor competence and physical self-perception and a correlation between fine motor skills and physical self-perception (r = −0.315).

The results show no correlations between motivation regulations and motor competence for the entire sample (Table 2), except for the correlation between heel-toe walking and intrinsic regulation (r = −0.216). In Table 3, the results show that there was only a correlation between heel-toe walking and identified regulation (r = −0.293) among boys. Among girls, there was a correlation between placing blocks and intrinsic regulation (r = −0.384).

There was a moderate association between physical self-perception and introjected regulation (r = 0.532) and intrinsic regulation (r = 0.555) in the whole sample, (Table 2). There was also a negative correlation with external regulation, which means that the lower self-perception the higher the external motivation. In Table 3, the results show that boys had a low-moderately correlation between physical self-perception and intrinsic and identified regulations. Moreover, there was a low correlation to introjected regulation (r = 0.282), but no correlation to external motivation. Among girls, there was a moderate correlation between physical self-perception and intrinsic (r = 0.593) and identified (r = 0.368) (Table 4). Unlike the boys, the girls had a negative relationship with the external regulation (r = −0.413). This means that the higher physical self-perception the more autonomous motivation and the lower physical self-perception the more external motivation.

## 4. Discussion

The results indicate low, but significant correlations between motor competence and physical self-perception throughout the sample. The entire sample and the sample divided by sex show significant correlation between autonomous motivation and physical self-perception. There were no significant correlations between autonomous motivation and motor competence in the sample.

The findings of a low correlation between physical self-perception and motor competence for the entire sample is in line with White’s [30] theory that people perceive their competence through effective interaction with the environment and Harter [27,28], who describes how children are attracted to what they master and have competence for. That boys have significant correlation to gross motor competence, while the girls have significant correlation to fine motor competence, can be explained by sex differences in terms of which activities are perceived as important. Although other domains of self-perception were measured in this study, other research has found that girls are influenced by preferences where fine motor skills are central. An example of this is Piek et al. [38], who point out that boys’ perceived motor skills have the strongest influence on physical self-perception and that academic self-perception does not play a crucial role. In girls, on the other hand, the connection between both athletic and academic self-perception is significant for perceived motor competence. This may mean that fine motor competence, which are more related to academic situations, are more important for girls than for boys.

There is a significant correlation between motor competence and physical self-perception for the entire sample and for girls, only. The results are in accordance with Vedul-Kjelsås [66], who reported significant correlation between motor competence and physical self-perception for the entire sample and for girls, but not for boys. Previous research shows that girls perform better in fine motor skills, while boys are better in gross motor skills [6,19,20,23,24,33,67]. In this study, no differences between sexes were found in motor competence measures. An explanation of the findings could be that perceived physical competence, as measured by SPPC, both captures aspect of different subdomains including sports, active play and motor skills [32]. Thus, boys can perceive themselves as physical competent independent of actual motor competence. Maturity and experience can be another possible explanation. The level of awareness of self-perception increases with maturity and age [6]. This may denote that girls, who have earlier onset of puberty than boys, have a greater awareness of their motor skills and athletic competence. Harter´s theory of competence motivation [27] is domain specific. Given that boys appear to be more physically active than girls [38], there is reason to believe that boys have higher gross motor skills than girls, due to the physical activities they engage in.

There is a clear association between physical self-perception and motivational regulation this in this study. The results for the entire sample show a significant connection between high physical self-perception and intrinsic and identified regulation for physical activity. The same is shown for both sexes separately. At the same time, the association between physical self-perception and external motivation, is significant for the entire sample and for girls. That is, children with higher self-perception display less extrinsic motivation (external regulation). It is therefore possible to argue that developing physical self-perception is essential to autonomous motivation stimulating to an active lifestyle and a lifelong joy in movement [61]. Although few studies have used the same questionnaires as in this study, the results are consistent with previous research in showing an association between autonomous motivation and physical self-perception or perceived physical competence in physical activity and physical exercise [46,52,68,69,70]. It is also interesting to note that items related to physical education scores lower on autonomous motivation compared to other physical activity. It may be that the quality of motivation decreases with age. Pure pleasure, via intrinsic motivation, tends to have a greater role in the participants before or in early puberty, and externally regulated duties and purposes appear to be of greater importance to those who are older.

In accordance with the self-determined continuum, the correlation output among the behavioral regulations display a “V” path; high and positive correlation between intrinsic and identified regulations and decreasing and negative correlations as the type of regulation is far away in the continuum. This is also found in the study by Estevan et al. [61]. Several studies have looked at how an autonomous learning environment in physical education increases students’ autonomous motivation [71,72,73]. In a review of motivation for physical exercise by Ntoumanis & Standage [68], one of the conclusions was that students should understand what positive effects, such as health effects, the activities in physical education have. Furthermore, the study concludes that SDT’s psychological needs should be promoted and that physical education teachers should give students choices, e.g., provide opportunities for choice of activities and input during the lessons [68]. At the same time, the effect of health on activities is one of the questions in the questionnaire BREQ [49] used in adults and in the revised version for children used in this study [46]. The question of valuing the activity falls under the category identified, i.e., autonomous motivation. Furthermore, health-related questions in the questionnaire are placed under controlled motivation: whether one feels unhealthy by not participating (introjected), and whether one participates because others say one should (external). This link may be a bit challenging for young students. The question here is whether students who are overweight, for example, should be pushed to participate through an argument about health effects. Such an argument can cause the motivation of vulnerable Student’s to move towards the external rather than the autonomous regulations. Motivation and physical self-perception must be considered as interacting factors when the aim is to change physical activity behavior [74]. Both boys and girls show lower physical self-perception and lower motivation for physical activity after the transition from childhood to adolescence [57]. The decrease in physical activity with age may be because physical activity and exercise feels more like a duty than pleasure.

The results from the entire sample show few significant correlations between motor competence and motivation. This is in line with Kalaja et al. [62], who found a cluster of participants who scored “high on motor skills/low on autonomous motivation” for physical activity. A possible explanation for high motor skills and low autonomous motivation for physical activity may be low perceived motor competence [61]. In the theories of competence motivation [27,75] and self-perception [28], competence is central to obtaining motivation for participation. However, the lack of clear correlations between these variables in this study, may be explained by the fact that physical self-perception does not necessarily correspond to actual motor competence as supported by the study of Meester et al. [60]. In the theory, emphasis is placed on the mental part of perception: what the person feels. This is described in Harter’s theory of self-perception [31], in terms of people’s actual qualities not having to correspond with reality. It is challenging to find clear associations between young children’s physical self-perception and actual motor competence, as physical self-perception often is characterized by effort and luck, and that they are unable to distinguish ability and effort [76]. Adolescents become more aware of their abilities and attributes with age, and thus assess their own competence as lower than they did in childhood [31]. From the age of 12, the results of Piek et al. [38] suggest that awareness of one’s own skills increases, therefore, with increasing age motivation can be more closely linked to actual motor competence [38]. Both actual motor competence, judgement about own competence and motivation changes during development, the relationship between the different variables also probably changes with age and gender during childhood. More longitudinal studies are needed in future research to see how these factors interact. Another explanation for these results is the inexistence of a linear correlation, as recent studies found curvilinear associations between actual, perceived MC and motivation [61].

A strength of this study is the inclusion of an objective measurement of motor skills. On the other hand, measuring motor behavior can be challenging and there is no defined gold standard for measuring motor skills [11,77]. The strength of TMC is that, unlike other motor tests designed to identify children with motor difficulties, it is sensitive at both ends of the scale, i.e., high and low motor competence [11]. Limitations of the study are that correlation analyses cannot reveal causal relationships between the different variables in addition to a relatively small sample size. Another limitation is the lack of person-centered analyses of the data as this could have expanded the understanding of, at the personal level, the association between perceived athletic competence and actual motor competence and motivation. Moreover, measurement of an individual’s motor competence is challenging, as it is a variable consisting of many different aspects and no gold standard measure exists to captures all aspects [78]. The TMC measures both aspects of gross- and fine motor competence, however, to find a measurable set of skills that can provide a representative picture of the general motor competence of an individual poses a significant challenge. Assessment of motor competence may also have led to a bias in the sample, as students who feel competent and confident in such a test situation wanted to participate, while those who may have felt little mastery in such a test situation refused to participate.

## 5. Conclusions

In this sample of 11–12-year-old children, no sex differences were found in motor competence measures, motivational regulations and physical self-perception. There is a clear association between the physical self-perception and autonomous motivation for the entire sample. The association between high physical self-perception and high autonomous motivation and at the same time low controlled motivation, suggests that it may be beneficial to create positive experiences and mastery for children. This can form the basis for high physical self-perception and autonomous motivation, which, in turn, can contribute to increased physical activity. Although no clear associations were found between motor competence and motivation, it is possible to argue that motor competence can influence motivation through other factors such as physical self-perception.

## Figures and Tables

**Table 1:** Descriptive statistics for age, sex, motor competence, physical self-perception and motivation for the entire sample (n = 101), boys (n = 53) and girls (n = 48).

Variable	Entire Sample		Boys		Girls		*p*-Value
	Mean	SD	Mean	SD	Mean	SD	
**Age**	11.74	0.57	11.85	0.50	11.61	0.62	
**Test of Motor competence**							
Placing bricks (seck)	24.67	2.72	25.29	2.97	24.05	2.47	0.031
Building bricks (seck)	12.82	2.01	13.05	1.83	12.60	2.20	0.188
Heel-to-toe walking (seck)	12.51	3.60	12.73	3.54	12.29	3.67	0.496
Running in slopes (seck)	5.34	0.55	5.23	0.05	5.46	0.55	0.048
**Motivation**							
Intrinsic	13.79	1.63	13.83	1.75	13.75	1.51	0.462
Identified	12.08	2.27	12.32	2.35	11.81	2.17	0.185
Introjected	9.21	2.15	9.47	2.49	8.92	1.69	0.255
External	4.87	1.91	5.09	1.92	4.63	1.89	0.167
**Physical self-perception**	3.05	0.50	3.13	0.49	2.97	0.51	0.151

**Table 2:** Correlation between motor competence, motivation and physical self-perception for the entire sample (n = 101).

	Whole Sample	1	2	3	4	5	6	7	8	9	10	11	12
1	TMC Z-score	1	0.803 **	0.811 **	0.637 **	0.744 **	0.563 **	0.737 **	−00.182	−0.087	−0.071	0.013	−0.242 *
2	Fine motor Z-score		1	0.358 **	0.829 **	0.883 **	0.225 *	0.350 **	−0.113	0.043	−0.020	−0.030	−0.085
3	Gross motor Z-score			1	0.263 **	0.349 **	0.726 **	0.852 **	−0.183	−0.160	−0.089	0.064	−0.282 **
4	Placing bricks				1	0.524 **	0.130	0.286 **	−0.193	0.079	−0.021	−0.019	−0.107
5	Building bricks					1	0.251 *	0.315 **	−0.087	−0.006	−0.023	0.039	−0.074
6	Heel-to-toe walking						1	0.311 **	−0.216 *	−0.172	−0.111	0.048	−0.141
7	Running in slopes							1	−0.096	−0.097	−0.059	0.041	−0.311 **
	**Motivation**												
8	Intrinsic								1	0.494 **	0.465 **	−0.237 *	0.555 **
9	Identified									1	0.491 **	0.037	0.532 **
10	Introjected										1	0.052	0.238 *
11	External											1	−0.308 **
12	Physical self-perception												1

** Correlation significant at the 0.01-level (2-tailed); * Correlation significant at the 0.05-level (2-tailed).

**Table 3:** Correlation between motor competence, motivation and physical self-perception in boys (n = 53).

	Boys	1	2	3	4	5	6	7	8	9	10	11	12
1	TMC Z-score	1	0.717 **	0.786 **	0.539 **	0.626 **	0.563 **	0.741 **	−0.076	−0.087	0.068	0.033	−0.190
2	Fine motor Z-score		1	0.197	0.796 **	0.864 **	0.107	0.226	−0.025	0.100	0.69	−0.045	0.089
3	Gross motor Z-score			1	0.116	0.164	0.763 **	0.856 **	−0.122	−0.180	0.027	0.131	−0.308 *
4	Placing bricks				1	0.454 **	0.098	0.151	−0.065	0.195	0.122	−0.050	0.111
5	Building bricks					1	0.102	0.151	−0.044	0.051	0.011	−0.028	0.101
6	Heel-to-toe walking						1	0.384 **	−0.264	−0.293 *	−0.117	0.028	−0.242
7	Running in slopes							1	0.044	−0.041	0.082	0.143	−0.316 *
	**Motivation**												
8	Intrinsic								1	0.577 **	0.501 **	−0.320 *	0.486 **
9	Identified									1	0.643 **	−0.064	0.638 **
10	Introjected										1	0.038	0.282 *
11	External											1	−0.253
12	Physical self-perception												1

** Correlation significant at the 0.01-level (2-tailed). * Correlation significant at the 0.05-level (2-tailed).

**Table 4:** Correlation between motor competence, motivation and physical self-perception in girls (n = 48).

	Girls	1	2	3	4	5	6	7	8	9	10	11	12
1	TMC Z-score	1	0.907 **	0.873 **	0.716 **	0.848 **	0.581 **	0.819 **	−0.284	−0.091	−0.243	−0.037	−0.315 *
2	Fine motor Z-score		1	0.631 **	0.837 **	0.895 **	0.353 *	0.671 **	−0.250	−0.110	−0.191	−0.067	−0.315 *
3	Gross motor Z-score			1	0.503 **	0.571 **	0.722 **	0.834 **	−0.235	−0.095	−0.223	0.008	−0.257
4	Placing bricks				1	0.567 **	0.150	0.604 **	−0.384 **	−0.101	−0.265	−0.062	−0.370 **
5	Building bricks					1	0.373 **	0.582 **	−0.175	−0.143	−0.128	0.058	−0.289 *
6	Heel-to-toe walking						1	0.290 *	−0.165	−0.056	−0.097	0.042	−0.090
7	Running in slopes							1	−0.236	−0.133	−0.254	−0.028	−0.252
	**Motivation**												
8	Intrinsic								1	0.360 *	0.417 **	−0.180	0.593 **
9	Identified									1	0.288 *	0.119	0.368 *
10	Introjected										1	0.025	0.149
11	External											1	−0.413 **
12	Physical self-perception												1

** Correlation significant at the 0.01-level (2-tailed). * Correlation significant at the 0.05-level (2-tailed).
